# The complete chloroplast genome of *Rubus lambertianus* var. *paykouangensis*, an edible wild plant

**DOI:** 10.1080/23802359.2022.2044399

**Published:** 2022-02-24

**Authors:** Ying-an Zhu, Shiyu Wang, Mingwei Hong, Shaojie Yuan, Xuehu Yang

**Affiliations:** a College of Landscape and Horticulture, Yunnan Agricultural University, Kunming, Yunnan, People’ Republic of China; b Alumni Association Office, Yunnan Agricultural University, Kunming, Yunnan, People’ Republic of China; c College of Tropical Crops, Yunnan Agricultural University, Pu-er, Yunnan, People’ Republic of China

**Keywords:** *Rubus lambertianus* var. *paykouangensis*, chloroplast genome sequence, Rosaceae, phylogeny

## Abstract

*Rubus lambertianus* Ser. var. *paykouangensis* (Levl.) Hand.-Mazz. is great important in the phylogeny and evolution of the genus *Rubus* L. in the family Rosaceae. The chloroplast genome of *R. lambertianus* var. *paykouangensis* reported in this study is 156177 bp in length, and it has an average GC content of 37.18%. The complete chloroplast genome showed a typical quadripartite structure, comprising a small single copy (SSC) region (18,730 bp) and a large single copy (LSC) region (85,883 bp), both of which were separated by a pair of inverted repeats (IRs, 25,782 bp). This plastome was discovered to contain 129 different genes (112 unique), including 85 protein-coding genes (79 unique), 36 tRNA genes (29 unique), and 8 rRNA genes (4 unique). The published chloroplast genome of *R. lambertianus* var. *paykouangensis* will provide a significant insight into elucidating the phylogenetic relationship of taxa within the genus *Rubus* of the family Rosaceae.

*Rubus lambertianus* Ser. var. *paykouangensis* (Levl.) Hand.-Mazz. belongs to genus *Rubus* L. section *Malachobatus* Focke, Subsection *Acuminati* (Focke) Yu et Lu in the family Rosaceae. It is widely distributed in Yunnan, Guizhou and Guangxi provinces of China (Robertson 1974; Yu and Lu 1985; Thompson 1995; Lu and Boufford 2003). It can be a very important fruit tree germplasm resource due to its fresh orange and delicious fruits. It is extremely difficult to distinguish the species from each other in the genus *Rubus* because of their complex morphological variations (Alice and Campbell 1999) due to their inter- and intraspecific hybridization, polyploidization and apomixis, especially between its original variant and the other two variants. In this study, we report the complete chloroplast genome of *R. lambertianus* var*. paykouangensis*, a wild species widespread in temperate, subtropical, and tropical zones in China, with elevations between 300 and 2200 m, as a molecular resource for future studies on the taxonomy of the genus *Rubus*.

The fresh leaves of *R. lambertianus* var*. paykouangensis* were collected from Cuihua Town, Daguan County, Zhaotong City, Yunnan Province of China (27°45′14″ N, 103°53′51″ E). A specimen was deposited at Herbarium of Horticultural Plants, Yunnan Agricultural University (https://www.ynau.edu.cn/, Shiyu Wang, 452060083@qq.com) under the voucher number Zhu-20201014R01. To construct chloroplast DNA libraries, total genomic DNA was extracted from fresh leaves using DNA Plantzol Reagent (Invitrogen, Carlsbad, CA, USA). The extracted DNA was sequenced by Illumina HiSeq Sequencing System (Illumina, San Diego, CA, USA) and a shotgun library was constructed. About 2.14 Gb pair-end (150 bp) raw reads were obtained, and the low-quality sequences were filtered by using CLC Genomics Workbench v8.0 (CLC Bio, Aarhus, Denmark) to produce high-quality clean reads. With the reference genome of *R. niveus* Thunb (MT576936), the complete chloroplast genome was aligned and assembled using NOVOPlasty software (Dierckxsens et al. 2017). The complete chloroplast genome of *R. lambertianus* var*. paykouangensis* was automatically annotated using CPGAVAS2 (Shi et al. 2019), and adjusted and confirmed with Geneious 9.1 (Kearse et al. 2012). Then we submitted the complete chloroplast genome to the GenBank database under the accession number of MZ352082. In order to ascertain the phylogenetic status of *R. lambertianus* var*. paykouangensis*, we used MAFFT (Katoh and Standley 2013) to align the complete chloroplast genome sequences of 35 species from three genera (6 species of *Fragaria*, 6 species of *Rosa*, and 23 species of *Rubus*) of the family Rosaceae. A maximum likelihood (ML) phylogenetic tree was reconstructed with the sequence alignments by RAxML8 (Stamatakis 2014) with GTR GAMMA I nucleotide model and 1000 bootstrap replicates.

The chloroplast genome of *R. lambertianus* var*. paykouangensis* is 156177 bp in size, and it has an average GC content of 37.18%. It showed a typical quadripartite structure comprising a small single-copy (SSC) region of 18,730 bp and a large single-copy (LSC) region of 85,883 bp separated by a pair of identical inverted repeat regions (IRs) of 25,782 bp each. The chloroplast genome is found to contain 129 genes (112 unique), including 85 protein-coding genes (79 unique), 36 tRNA genes (29 unique), and 8 rRNA genes (4 unique).

The ML phylogenetic tree shows that *R. lambertianus* var*. paykouangensis* is mostly related to *R. lambertianus* var*. glaber* in the genus *Rubus*, with bootstrap support values of 100% (Figure 1). All species of the other two genera (*Rosa* and *Fragaria*) from the family Rosaceae have been formed into an independent monophyly. The monophyly of three genera of the family Rosaceae was well-supported by using the complete chloroplast genome sequences. This research lays the foundation for further understanding of the chloroplast genome information of the genus *Rubus*, and sets a new insight into clarifying the phylogeny and genomics of the family Rosaceae.

**Figure 1.**
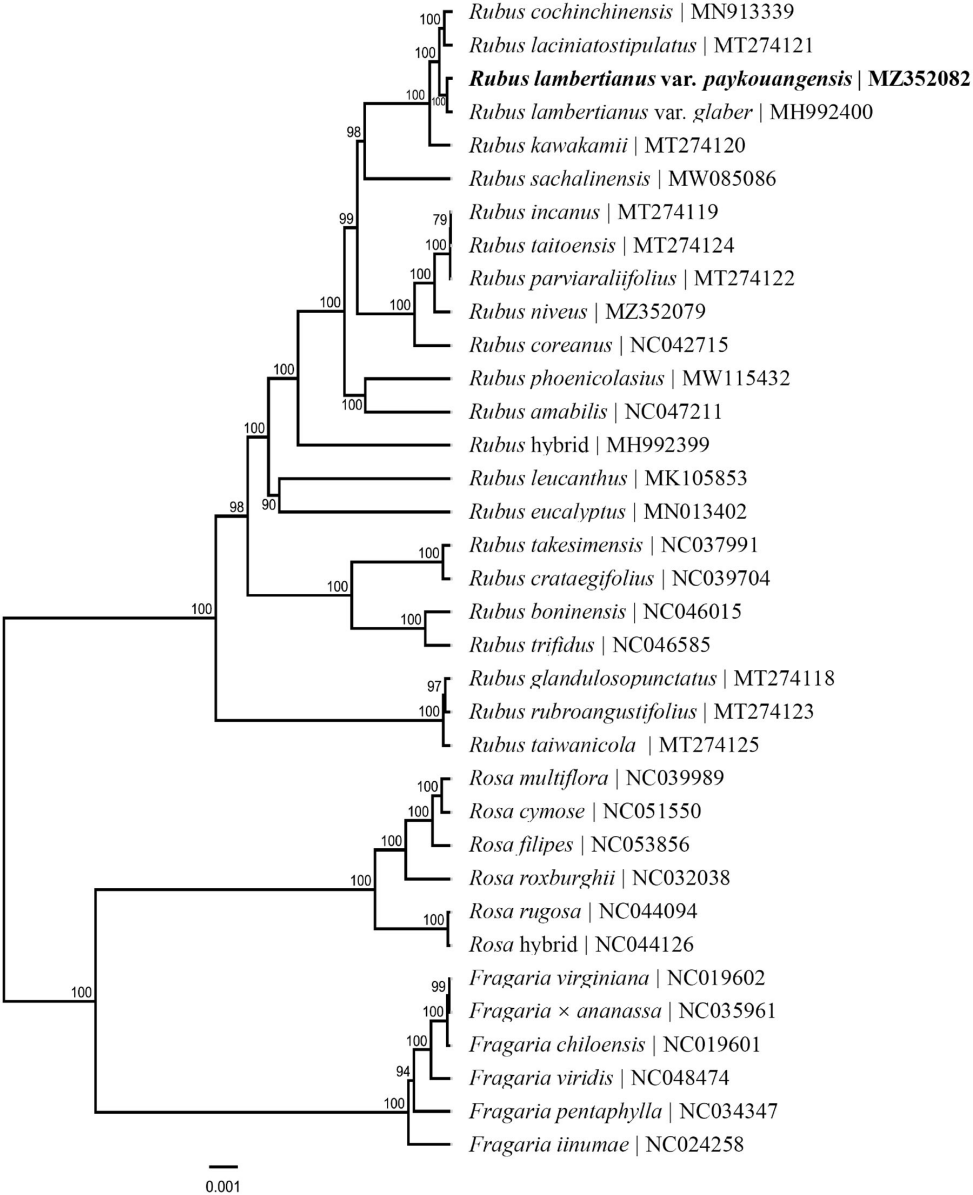
Phylogenetic relationships of 35 species from three genera of *Fragaria*, *Rosa*, and *Rubus* in the family Rosaceae based on the complete chloroplast genome sequences. Bootstrap percentages are indicated for each branch.

## Author contributions

Ying-an Zhu, Shaojie Yuan, and Xuehu Yang were involved in the conception and design, Shiyu Wang and Mingwei Hong analyzed and interpreted the data; Ying-an Zhu drafted the paper, Shaojie Yuan and Xuehu Yang revised it critically for intellectual content; all authors approved the final version to be published; and agreed to be accountable for all aspects of the work.

## Data Availability

The complete chloroplast genome generated for this study has been deposited in GenBank under accession number MZ352082, which is openly available in GenBank through the NCBI at website (https://www.ncbi.nlm.nih.gov/). All high-throughput sequencing data files are available from the GenBank Sequence Read Archive (SRA) under accession number SRR14757447. The associated BioProject and Bio-Sample numbers are PRJNA735803 and SAMN19602745, respectively.
